# The refractive state of the eye in Icelandic horses with the *Silver* mutation

**DOI:** 10.1186/s12917-017-1059-7

**Published:** 2017-06-02

**Authors:** Maria K. Johansson, Kim Jäderkvist Fegraeus, Gabriella Lindgren, Björn Ekesten

**Affiliations:** 1 Department of Animal Breeding and Genetics, Swedish University of Agricultural Sciences, -750 07 Uppsala, SE Sweden; 2 Department of Clinical Sciences, Swedish University of Agricultural Sciences, -750 07 Uppsala, SE Sweden

**Keywords:** Multiple Congenital Ocular Anomalies, MCOA, Refraction, Myopia, Skiascopy, Horse -Icelandic horses

## Abstract

**Background:**

The syndrome Multiple Congenital Ocular Anomalies (MCOA) is a congenital eye disorder in horses. Both the MCOA syndrome and the Silver coat colour in horses are caused by the same missense mutation in the *premelanosome protein* (*PMEL)* gene*.* Horses homozygous for the *Silver* mutation (TT) are affected by multiple ocular defects causing visual impairment or blindness. Horses heterozygous for the *Silver* mutation (CT) have less severe clinical signs, usually cysts arising from the ciliary body iris or retina temporally. It is still unknown if the vision is impaired in horses heterozygous for the *Silver* mutation. A recent study reported that Comtois horses carrying the *Silver* mutation had significantly deeper anterior chambers of the eye compared to wild-type horses. This could potentially cause refractive errors. The purpose of the present study was to investigate if Icelandic horses with the *Silver* mutation have refractive errors compared to wild-type horses. One hundred and fifty-two Icelandic horses were included in the study, 71 CT horses and five TT horses. All horses were genotyped for the missense mutation in *PMEL*. Each CT and TT horse was matched by a wild-type (CC) horse of the same age ± 1 year. Skiascopy and a brief ophthalmic examination were performed in all horses. Association between refraction and age, eye, genotype and sex was tested by linear mixed-effect model analysis. TT horses with controls were not included in the statistical analyses as they were too few.

**Results:**

The interaction between age and genotype had a significant impact on the refractive state (*P* = 0.0001). CT horses older than 16 years were on average more myopic than wild-type horses of the same age. No difference in the refractive state could be observed between genotypes (CT and CC) in horses younger than 16 years. TT horses were myopic (−2 D or more) in one or both eyes regardless of age.

**Conclusion:**

Our results indicate that an elderly Icelandic horse (older than 16 years) carrying the *Silver* mutation is more likely to be myopic than a wild-type horse of the same age.

**Electronic supplementary material:**

The online version of this article (doi:10.1186/s12917-017-1059-7) contains supplementary material, which is available to authorized users.

## Background

The syndrome Multiple Congenital Ocular Anomalies (MCOA) is a congenital eye disorder in horses. It was first described in Rocky Mountain Horses in 1999 [1]. Previous studies have shown that both the Silver coat colour and the MCOA syndrome in horses are caused by the same missense mutation in the *premelanosome protein* (*PMEL)* gene [2, 3]. The mutation, a change from cytosine (C) to thymine (T), causes a dilution of the eumelanin pigment especially in the mane and tail [2]. *PMEL* encodes the transmembrane glycoprotein PMEL also known as PMEL17, which is essential for the biogenesis of eumelanin in the melanosomes. PMEL forms fibrillary structures where melanin is deposited during melanogenesis [4, 5]. Post translational and proteolytic processing of PMEL are required for a correct formation of the fibrillar matrix. It is not clear how these processes are regulated [6, 7]. In a study from 2011 a knock-out mouse line where *PMEL* had been inactivated was created [8]. It was shown that PMEL was important for eumelanin production in skin, choroid and retinal pigment epithelium. However, full-field electroretinogram did not show any impaired function of the retina [8].

Clinically, Silver coloured horses can be subdivided into two different groups, the Cyst phenotype and the MCOA phenotype, according to the severity of the ocular defects. Horses with the Cyst phenotype are known to be heterozygous for the *Silver* mutation (CT). They have less severe ocular signs predominantly iridociliary cysts, usually in the temporal quadrant of the eye. The cysts are translucent and can be up to a centimetre in size. Horses with the MCOA phenotype are known to be homozygous for the *Silver* mutation (TT). They have more severe clinical signs including iridal stromal hypoplasia, miotic pupils, retinal dysplasia, cataract, cornea globosa and iridociliary cysts occasionally extending subretinally [1, 9–11]. An incomplete dominant mode of inheritance has been suggested [10, 12, 13].

The ocular defects that are present in horses homozygous for the *Silver* mutation cause visual impairment or blindness [1, 9–11]. It is still unknown if vision is impaired in horses heterozygous for the *Silver* mutation. Hence, more information is needed to investigate how the MCOA syndrome affects the vision in horses heterozygous for the *Silver* mutation. A recent study in Comtois and Rocky Mountain horses reported that horses with the *Silver* mutation, both heterozygotes and homozygotes, had deeper anterior chambers of the eye compared to wild-type horses. A significant difference in the depth of the chamber was also detected between heterozygous and homozygous horses [11]. An abnormal distance between the refractive surfaces of the eye is likely to cause refractive errors. The purpose of the present study was to investigate if Icelandic horses with the *Silver* mutation have refractive errors compared to wild-type horses of the same breed.

## Methods

### Animals

**Fig. 1:**
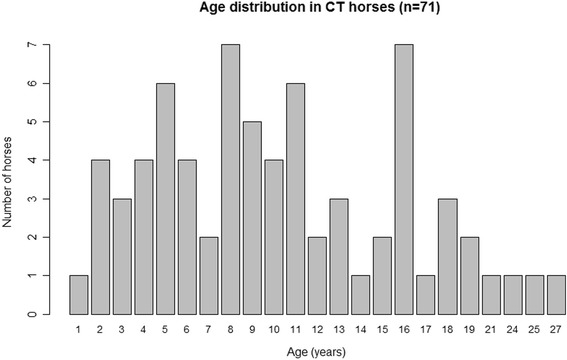
The age distribution of CT horses (*n* = 71)

**Fig. 2:**
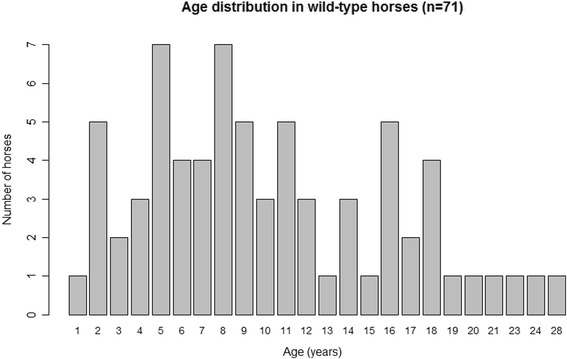
The age distribution in controls (wild-type) horses (*n* = 71)

In total 152 Icelandic horses were included in the study, 71 were heterozygous and five homozygous for the *Silver* mutation. Each CT and TT horse was matched by a control (wild-type) horse of the same breed and age ± 1 year. Control horses were confirmed being wild-type (CC) for both disease-alleles. In the CT group was the median age of both CT and controls 10.3 years, ranging from 1 to 27 years in CT and from 1 to 28 years in control horses (Figs. 1 and 2). The median age of the TT horses was 11.4 years and 11.8 years in their age-matched controls with a range from 1 to 22 years in both TT and control horses. The sex distribution in the CT horse group was 33 males and 38 females in CT horses and 24 males and 47 females in controls. In the TT group one male and four females were compared to four males and one female controls. All horses in the study were enrolled after posting information on social media and by contacting horse farms near Uppsala, Sweden.

### Phenotypic assessment

Phenotypic data on all horses in the study were obtained from a brief ophthalmic examination and skiascopy without dilation of the pupil. The ophthalmic examination was performed in the stable under dim-light conditions. The examination included testing of direct and indirect pupillary light reflexes, dazzle reflex and menace response to crudely assess the function of the retina and the optic nerve. Palpebral and corneal reflexes were tested to control the function of the sensory innervation of the eye. The reflexes were all graded as normal, decreased or absent. If the reflex was decreased it was described as being either slow or incomplete. All eyes were scanned for opacities in the ocular media visible to the examiner’s naked eye with the retinoscope as a light source. Opacities, if present, were graded based on extension and location. The cornea was observed to find signs of obvious cornea globosa. Iridociliary cysts or other anomalies were also noted. The exam record used in this study can be found in Additional file 1.

Skiascopy was performed with a streak retinoscope (Heine Beta 200) and Trousseau racks without dilation of the pupils. Lenses in steps of 0.5 D were used to reach the neutralization point and the working distance was subtracted. The refractive power was measured both horizontally and vertically in both eyes. Astigmatism was defined as a difference of more than 0.5 D between the horizontal and vertical meridians.

### DNA isolation and genotyping

Genomic DNA was isolated from hair samples by the standard hair-preparation method as previously described [14]. All horses in the study were genotyped for the missense mutation in *PMEL* previously shown to be associated with both the Silver coat colour and the MCOA syndrome [2, 3]. The genotyping was performed by using custom designed TaqMan SNP Genotyping Assays (Applied Biosystems StepOnePlus™ Instrument by Life Technologies) for SNP g.73665304 on chromosome 6 [3]. Horses were classified according to the genotype into three groups; wild-type (CC), heterozygous (CT) and homozygous (TT) for the *PMEL* missense mutation.

### Statistical analysis

All statistical analyses were performed in the software environment R-3.1.2 (R Development Core Team, 2014) and significance was defined as *P* ≤ 0.05. TT horses were not included in the statistical analyses because of very small sample size.

Association between refraction and age, eye, genotype and sex was tested by linear mixed-effect model analysis with the function lmer and the packages lme4 and car. There were two observations per horse, one for each eye (right and left) and the model used in lme4 was:

lmer(refraction ~ (age + eye + genotype + sex)^2 + (1|horse), data = data.

The fixed factors in the model; age (age of the horse at the examination in years), eye (right or left), genotype (CT or CC) and sex (male or female) were defined as factor variables. Pairwise interactions between each fixed factor were included in the model. The identity of the horse was used as a random factor. The dependent variable refraction (the horizontal refractive value determined by skiascopy) was defined as a continuous numeric variable. A Wald chi-square test was used in the post hoc test with Anova type 3 to estimate *P*-values. Differences in refraction related to genotype and age were further tested in a post hoc test with the function least-square means (lsmeans). Horses were divided into two-year age-classes and pairwise comparisons were calculated.

## Results

### Heterozygous (CT) horses

**Fig. 3:**
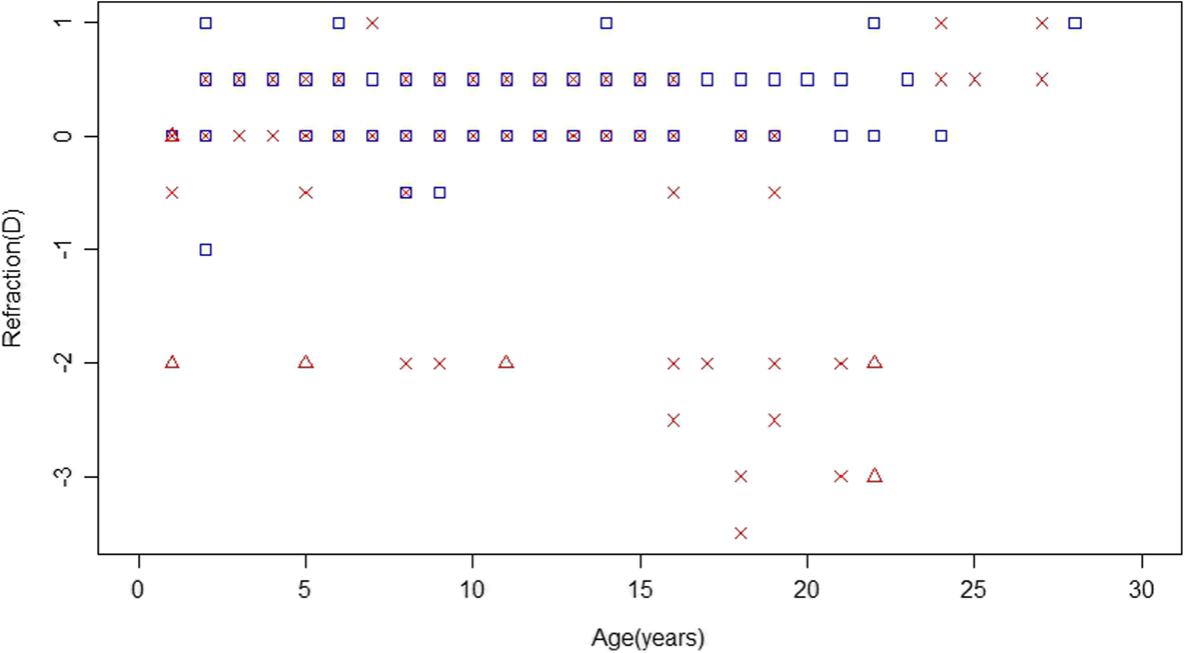
The refraction values according to age in all 152 Icelandic horses. *Blue* squares indicate wild-type horses, *red crosses* CT horses and *red* triangles TT horses. (Notice that some horses of the same age have the same refraction value and therefore some data points are superimposed on each other). Refraction values for both right and left eye are presented for each of the 152 horses

The mean refractive value ± SE was −0.09 ± 0.10 D for the right eye and 0.0 ± 0.09 D for the left eye in CT horses compared to 0.26 ± 0.04 D for right and 0.28 ± 0.04D for left eye in wild-type horses. The refractive state of the majority of both CT and wild-type horses was close to 0 D, although the average wild-type Icelandic horse was slightly hyperopic (farsighted) (Table 1). None of the horses were astigmatic according to the definition used in the study. Two CT horses, 18 and 21 years old, had severe refractive errors (−3 D or more) in one or both eyes (Fig. 3). The interaction between age and genotype had a significant impact on the refractive state (*P* = 0.0001). No other interactions tested were statistically significant. The refractive state was significantly different between genotypes (CT and CC) in ages 16–21 years. CT horses older than 16 years were on average more myopic compared to CC horses of the same age. The *P* value was 0.003 for age group 16–17 years, 0.0001 for age group 18–19 years and <0.0001 for age group 20–21 years. Horses 22 years and older could not be analysed because they were too few.

**Table 1:** The refractive state of all 152 horses for right and left eye respectively

Right eye	Number of CT horses	Number of TT horses^a^	Number of CC horses
**Emmetropic (normal sighted) 0 D**	37 (52.1%)	0 (0%)	32 (42.1%)
**Hyperopic (farsighted)**			
Slightly (+0,5–1 D)	24 (33.8%)	0 (0%)	41 (53.9%)
Moderate (+1,5–2,5 D)	0 (0%)	0 (0%)	0 (0%)
Severe > +3 D	0 (0%)	0 (0%)	0 (0%)
**Myopic (nearsighted)**			
Slightly (−0,5–1 D)	3 (4.2%)	0 (0%)	3 (3.9%)
Moderate (−1,5–2,5 D)	5 (7.0%)	3 (75%)	0 (0%)
Severe (−3 D or more)	2 (2.8%)	1 (25%)	0 (0%)
Left eye	Number of CT horses	Number of TT horses^a^	Number of CC horses
**Emmetropic (normal sighted) 0 D**	37 (52.1%)	1 (25%)	29 (38.2%)
**Hyperopic (farsighted)**			
Slightly (+0,5–1 D)	25 (35.2%)	0 (0%)	44 (57.9%)
Moderate (+1,5–2,5 D)	0 (0%)	0 (0%)	0 (0%)
Severe > +3 D	0 (0%)	0 (0%)	0 (0%)
**Myopic (nearsighted)**			
Slightly (−0,5–1 D)	3 (4.2%)	0 (0%)	3 (3.9%)
Moderate (−1,5–2,5 D)	5 (7.0%)	3 (75%)	0 (0%)
Severe (−3 D or more)	1 (1.4%)	0 (0%)	0 (0%)

^a^Notice that sciascopy could not be performed in one of the TT horses because of miotic pupils and dense cataract on both eyes

Only one CT horse in the study had an immature cataract visible to the naked eye. It affected about 25% of the area of the lens in the coronal plane in the right eye. This horse was also myopic (−3 D in the right eye and −2 D in the left eye). Eight CT horses had mild cornea globosa and two CT horses had moderate cornea globosa in one or both eyes. A 25 year old CT horse had moderately miotic pupils and the pupillary reflexes were abnormal in both eyes. All CT horses in the study, except the horse with miotic pupils, had normal reflexes. Twenty-three CT horses of different ages had temporal cysts in one or both eyes that were visible to the naked eye. The cysts ranged in diameter up to a centimetre. No correlation between age and presence of cysts was observed.

### Homozygous (TT) horses

The mean refractive value ± SE was −2.25 ± 0.22 D for the right eye and −1.50 ± 0.43 D for the left eye in TT horses compared to 0.40 ± 0.17 D for right and 0.20 ± 0.11 D for left eye in wild-type horses. One 18 years old TT horse had dense cataract, severely miotic pupils and absent pupillary light reflexes in both eyes. Skiascopy could not be performed in this horse. All the other TT horses in the study had mild to moderate cornea globosa and moderately miotic pupils with a decreased and incomplete pupillary light reflexes in both eyes. All four TT horses were myopic (−2 D or more) in one or both eyes (Table 1). The presence of cysts and minor, peripheral cataracts was difficult to assess in the TT horses because of their miotic pupils.

## Discussion

This study was performed to provide additional insight into how the MCOA syndrome affects the vision in horses heterozygous for the *Silver* mutation. Our results indicate that horses older than 16 years carrying the *Silver* mutation are more likely to be myopic than wild-type horses of the same age. In the present study CT horses older than 16 years on average were more myopic than wild-type horses of the same age. No difference in the refractive state could be observed between genotypes (CT and CC) in horses younger than 16 years.

A myopic shift in elderly individuals has also been observed in human beings and dogs [15–17]. Although humans experience a hyperopic shift with age as they develop presbyopia, a long-term study has shown that this trend reverses in the direction of myopia in the oldest group (70 years and older) [15]. To our knowledge, there are few studies on how the refractive state changes with age in horses. A degree project at the Swedish University of Agricultural Sciences by Löf from 2007, where 116 Standardbred trotters were included in a cross-sectional study, showed that elderly horses had a progression towards myopia. In the current study we observed a significant difference in the refractive state between CT and CC horses in ages 16–21 years. Horses older than 21 years could not be further analysed because of small sample size. Seven of the CT horses had moderate or severe myopia (−1.5 D or more) and five of these were 16 years or older. These horses were not closely related and they came from different stables.

The observed shift towards myopia in elderly CT horses may also suggest that the *Silver* mutation exerts a slowly progressive effect on the optics of the eye. However, one important limitation is that this was a cross-sectional study and we did not have the possibility to follow these horses from when they were foals up to old age. The MCOA syndrome is generally thought to be non-progressive [1], although there are indications that at least some clinical signs may progress [11]. The study by Ségard et al. from 2013, where 59 Comtois and 16 Rocky Mountain horses ranging from 10 days to 18 years of age were included, reported no differences in presence of ocular abnormalities between young, adult and old horses for the majority of the defects detected [11]. However, a possible increase in the number of cystic lesions with age was suggested. Foals with the *Silver* mutation, both homozygotes and heterozygotes, had significantly fewer cysts compared to adult and old horses of the same genotype [11]. The study by Plummer and Ramsey from 2011 [18], where 53 American Miniature horses were included, showed no correlation between age and presence of ocular abnormalities. The median age of the horses in that study was 5.3 years with a range from 1.5 to 219 months (18.25 years). Hence, the median age of the horses was lower in the previous studies [11, 18] compared to our study. In our study, 23 (32%) of the CT horses of different ages had visible cysts in one or both eyes. No correlation between age and presence of cysts or other ocular abnormalities could be observed. Cysts were hard to detect in TT horses because of miotic pupils. Most likely both CT and TT horses had cysts that could not be detected because we neither dilated the pupil, nor used more sensitive methods such as ultrasonography to evaluate the presence of cysts. However, that was beyond the scope of this study.

We found no difference in the refractive state between genotypes (CT and CC) in horses younger than 16 years. One possible reason could be that the refractive errors were minimized by other, compensatory mechanisms. Therefore it would have been interesting to measure other parameters, including anterior chamber depth and axial length that could shed light on the underlying causes of the refractive results of the horses in our study. Unfortunately, the ophthalmic examinations were performed in stables and no equipment for precise measuring of additional ocular biometric parameters was available.

Only five TT horses were included in our study. These horses were difficult to find, probably because of a low frequency of the TT genotype, as horse owners are recommended not to mate two Silver coloured horses. TT horses had more severe ocular signs than CT horses, which is in accordance with previous studies [1, 9–11]. These horses also represented a special challenge to refract because of their miotic pupils. Skiascopy could not be performed in one of the TT horses because of cataractous lenses in combination with very miotic pupils, two clinical signs already described as being part of the MCOA phenotype [1, 9–11]. TT horses were myopic (−2 D or more) in one or both eyes regardless of age. Only one CT horse in the study had an immature cataract visible to the naked eye. This horse was also myopic (−3 D in the right eye and −2 D in the left eye). Some individuals, both CT and TT, seem to have more severe clinical signs of the MCOA syndrome. Hence, additional genetic factors may be involved. None of the wild-type horses in the study were myopic (−1.5 D or more). The Icelandic horse as a breed seems to be slightly hyperopic on average. This is in accordance with the results from a degree project at the Swedish University of Agricultural Sciences by Östberg in 2007, where 26 Icelandic horses with a median age of 11.5 years were included.

Eye (left or right) did not have a significant impact on the refractive state. This was as expected since the MCOA syndrome is reported to affect the eyes bilaterally [1]. The previous study by Ségard et al. from 2013 reported no significant difference in depth of the anterior chamber between right and left eye [11]. Sex was also not found to affect the refractive state in the present study. This was in accordance with previous studies [19, 20].

The horses in the present study were examined without dilation of the pupils. Previous studies have shown that cycloplegia has no impact on streak retinoscopic evaluation of refraction [19, 21]. Two degree projects at the Swedish University of Agricultural Sciences from 2007 by Östberg and Löf also showed that cycloplegia is not needed before retinoscopy in horses older than 9 months.

We considered the horizontal refractive values most convenient to obtain because of the ellipsoid shape of the pupil in horses and therefore most accurate to assess. Hence, only the horizontal values were used in the statistical analyses. However, the differences between the horizontal and vertical values were less than 0.5 D in all horses and therefore none of the horses were considered to be astigmatic.

One of the major strengths of our study is that we have a large dataset that represents a wide range of ages in a homogenous population with only one breed. Each CT and TT horse was well matched by a wild-type horse of the same breed and age ± 1 year.

This study provides valuable information about the refractive state in Icelandic horses with the *Silver* mutation which can be used in future breeding recommendations. Horse owners should be aware that Silver colored horses older than about 16 years may have an increased risk to become myopic compared with wild-type horses.

## Conclusion

Our results indicate that horses older than 16 years carrying the *Silver* mutation are more likely to be myopic than wild-type horses of the same age.

## Additional files

Additional file 1: Medical record for the ophthalmic examinations (DOCX 12 kb)

Additional file 2: Tabular data generated during this study can be found in Additional file 2. (XLSX 20 kb)

## Abbreviations

MCOA: Multiple Congenital Ocular Anomalies
PMEL: Premelanosome protein
SE: Standard error
SNP: Single nucleotide polymorphism
