# Erratum to: The refractive state of the eye in Icelandic horses with the *Silver* mutation

**DOI:** 10.1186/s12917-017-1111-7

**Published:** 2017-06-29

**Authors:** Maria K. Johansson, Kim Jäderkvist Fegraeus, Gabriella Lindgren, Björn Ekesten

**Affiliations:** 1 Department of Animal Breeding and Genetics, Swedish University of Agricultural Sciences, 750 07 Uppsala, SE Sweden; 2 Department of Clinical Sciences, Swedish University of Agricultural Sciences, 750 07 Uppsala, SE Sweden

## Erratum

Due to an error carried forward by the Production team handling this article, the original article [1] contained a major discrepancy whereby the Figures were presented in the incorrect order.

Consequently, the original article has now been corrected to display the Figures in the correct order.
