# Supplementary material for: The refractive state of the eye in Icelandic horses with the Silver mutation
Source: BMC Vet Res. 2017 Jun 2;13:153. doi: 10.1186/s12917-017-1059-7 (PMC5455091; doi:10.1186/s12917-017-1059-7)
Supplement: Supplementary file 1 — Medical record for the ophthalmic examinations (DOCX 12 kb) [file 12917_2017_1059_MOESM1_ESM.docx]

**Medical record for ophthalmic examination MCOA 2016**

**Horse ID:**

Name:

ID number:

Sex:

Breed:

Color:

Date of birth:

Owner:

**Date**:

**Anamnesis:**

**Reflexes:** (normal/decreased (slow or incomplete) /absent)

**Right eye:**

Direct pupillary light reflex:

Indirect pupillary light reflex:

Menace response:

Palpebral reflex:

Corneal reflex:

Dazzle reflex:

**Left eye:**

Direct pupillary light reflex:

Indirect pupillary light reflex:

Menace response:

Palpebral reflex:

Corneal reflex:

Dazzle reflex:

**Ophthalmic examination**

**Right eye:**

Cornea: shape, if any signs of cornea globosa (with the light from the ophthalmoscope)

Other visual anomalies including cysts (with the light from the ophthalmoscope):

**Left eye:**

Cornea: shape, if any signs of cornea globosa (with the light from the ophthalmoscope)

Other visual anomalies including cysts (with the light from the ophthalmoscope):

**Skiascopy right eye:**

Horizontally:

Vertically:

Astigmatism (yes/no):

Fundus reflex (neutralization point):

Cataract: absent, + (mild), ++ (moderate) or +++ (abundant)

**Skiascopy left eye:**

Horizontally:

Vertically:

Astigmatism (yes/no):

Fundus reflex (neutralization point):

Cataract: absent, + (mild), ++ (moderate) or +++ (abundant)

Definition of the cataract

Location: Nuclear or cortical

Extension:

Absent: No visible cataract.

Mild: cataract extending over less than 25 % of the area of the lens in the coronal plane.

Moderate: cataract extending over 25 %, but less than 50 % of the area of the lens in the coronal plane.

Abundant: cataract extending over at least 50 % of the area of the lens in the coronal plane.
